# Stenotic intercondylar notch is not a risk factor for posterior cruciate ligament rupture: a morphological analyses using magnetic resonance imaging

**DOI:** 10.1007/s00167-021-06724-3

**Published:** 2021-09-02

**Authors:** Fei Liu, Sheng Zhang, Yang Xiao, Xiaoreng Feng, Zhenming Liang, Frankie Leung, Bin Chen

**Affiliations:** 1 Division of Orthopaedics and Traumatology, Department of Orthopaedics, Nanfang Hospital, Southern Medical University, No. 1838 North Guangzhou Avenue, 510515 Guangzhou, China; 2 Department of Orthopaedics, People’s Hospital of Hua Zhou, Maoming, 525100 China; 3 Department of Orthopaedics and Traumatology, Queen Mary Hospital, The University of Hong Kong, Hong Kong, China; 4 Department of Joint Surgery and Sports Medicine, Center for Orthopaedics Surgery, The Third Affiliated Hospital of Southern Medical University, Guangzhou, 510630 China; 5 Department of Orthopaedics and Traumatology, Yangjiang People’s Hospital, Yangjiang, 529535 China

**Keywords:** Posterior cruciate ligament rupture, Morphological characteristics, Magnetic resonance imaging

## Abstract

**Purpose:**

The present study aimed to examine the factors related to the morphological characteristics of the femoral condyle in posterior cruciate ligament rupture in female and male populations.

**Methods:**

One hundred and three patients (41 females, 62 males) with posterior cruciate ligament rupture from 2010 to 2020 were included in this retrospective case–control study. The sex and age of the posterior cruciate ligament rupture group were matched to those of the control group (41 females, 62 males; age range 16–69 years). Magnetic resonance imaging was used to measure the intercondylar notch width, femoral condylar width, and intercondylar notch angle in both the axial and coronal images. The ‘α’ angle was also measured using magnetic resonance imaging. The notch width index is the ratio of the intercondylar notch width to the femoral condylar width. Three types of intercondylar notch shapes (types A, U, and W) were evaluated in the axial magnetic resonance imaging images.

**Results:**

The difference in the mean coronal notch width index between the study groups was statistically significant in the female population. The difference in the mean coronal femoral condylar width between the study groups was statistically significant in the male population.

**Conclusions:**

A larger coronal notch width index was the greatest risk factor for posterior cruciate ligament rupture in the female population. In the male population, decreased coronal condylar width was the greatest risk factor for posterior cruciate ligament rupture. The results did not indicate that patients with a PCL rupture have a stenotic intercondylar notch. Posterior cruciate ligament injury prevention strategies could be applied to females with a larger coronal notch width index and males with a decreased condylar width.

**Levels of evidence:**

Level III.

## Introduction

The posterior cruciate ligament (PCL) is a strong ligament that prevents the tibia from moving backward excessively [18]. Thus, PCL rupture is often caused by direct posterior forces. In developed countries, young males (approximately 30 years old) are the main population group affected by PCL rupture [16]. Among football players, approximately 5% of knee injuries involve the PCL [17]. The acute phase of PCL rupture can lead to knee pain and functional limitations. As the condition progresses, > 10% of conservatively treated patients might develop moderate to severe traumatic osteoarthritis [20].

At present, many studies have assessed the morphological risk factors for anterior cruciate ligament (ACL) rupture. Risk factors for ACL rupture include a narrow intercondylar notch, lower notch width index (NWI), a smaller tibial eminence, a stenotic intercondylar notch shape, and increased tibial slope [2, 3, 5, 7, 12, 14]. The results of these studies can help individuals with these risk factors prevent ACL injuries during exercise [5]. The increased tibial slope is related to ACL injuries as the increased tibial slope can cause a greater anterior displacement of the tibia in sports [10, 12, 13]. In contrast, a decreased tibial slope is a risk factor for PCL rupture because an increased tibial slope can better prevent the tibia from moving backward excessively and avoid PCL injury [4, 9]. In a retrospective case–control study, Bernhardson et al. found that decreased posterior tibial slope was a risk factor for PCL rupture based on measurements on lateral radiographs of the knee, as the posterior tibial slope of patients with PCL injury was approximately 6° smaller than that in the control group [4]. Therefore, the risk factors for ACL injury are not necessarily also risk factors for PCL injury and might even prove protective factors against PCL injury. Van Kuijk et al. established statistical shape models of the knee using anteroposterior (AP), lateral, and Rosenberg radiographic views. The results showed that the intercondylar notch was smaller and more sharply angled, and the tibial eminence was more flattened in patients with PCL rupture [22]. Nevertheless, studies on the morphological risk factors for PCL rupture remain limited due to the low incidence of PCL rupture. The morphological characteristics of the knee that might be related to PCL injury remain to be further studied.

The femoral condyle is a complex three-dimensional structure. Morphological measurements are widely used to study the related anatomical characteristics of knee sports injuries, especially ACL injuries. At present, the most commonly used measurement indicators for ACL injury include the ‘α’ angle value, femoral condylar width, intercondylar notch width, NWI, intercondylar notch angle, and intercondylar notch shape [2]. However, the relationship between these morphological features and PCL injury is unclear and might not be the same as that noted for ACL injury.

The present study aimed to explore the relationship between the morphological characteristics of the femoral condyle and PCL rupture using magnetic resonance imaging (MRI). The morphological parameters of the femoral condyle include the ‘α’ angle value, femoral condylar width, intercondylar notch width, NWI, intercondylar notch angle, and intercondylar notch shape. The authors hypothesize that decreased femoral condylar width and narrow intercondylar notch width are the most important risk factors for PCL rupture. The association of PCL rupture with other morphological parameters, including NWI, ‘α’ angle value, intercondylar notch angle, and intercondylar notch shape, was also evaluated.

## Materials and methods

The procedures of this study complied with the Declaration of Helsinki and the relevant Chinese policies. The study protocol was approved by the Ethical Review Committee of People's Hospital of Hua Zhou. The IRB number is PJ202000301.

One hundred and three patients (41 females, 62 males) met the inclusion criteria for the PCL rupture group in this study. The control group of 103 patients (41 females, 62 males) was matched by sex and age to the case group. The mean (SD; range) ages of the PCL rupture group and the control group were 38.2 (13.8; 16–67) years old and 38.4 (13.8; 16–69) years old, respectively. All patients underwent MRI examination of the unilateral or bilateral knee joint. The images were obtained using a Philips Ingenia 3.0 Tesla MRI scanner (Royal Philips, Amsterdam, Netherlands). Knee imaging protocol included five sequences with the following parameters: *Coronal T1w*: TR: 569 ms, TE: 20 ms, 3.5-mm slice thickness/0.35-mm gap, matrix: 384 × 312, *Coronal T2spair*: TR: 2078 ms, TE: 65 ms, 3.5-mm slice thickness/0.35-mm gap, matrix: 308 × 240, *Sagittal T1w*: TR: 569 ms, TE: 20 ms, 3.5-mm slice thickness/0.8-mm gap, matrix: 384 × 311, *Sagittal T2spair*: TR: 1963 ms, TE: 62 ms, 3.5-mm slice thickness/0.8-mm gap, matrix: 308 × 240, *Transverse T2spair*: TR: 2079 ms, TE: 65 ms, 4-mm slice thickness/0.4-mm gap, matrix: 292 × 210. Patients with obvious radiological signs of osteoarthritis, including deformation and loss of cartilage in the knee joint, bone regeneration of the joint margin, and subchondral bone, were excluded. The PCL rupture group consisted of patients with a partial or complete rupture of the PCL but intact ACL as diagnosed by an MRI or arthroscopy examination. The control group consisted of patients with collateral ligament injury, chondral lesions, or meniscal tears due to a knee injury with no injuries to the PCL and ACL based on MRI or arthroscopy examination.

**Fig. 1:**
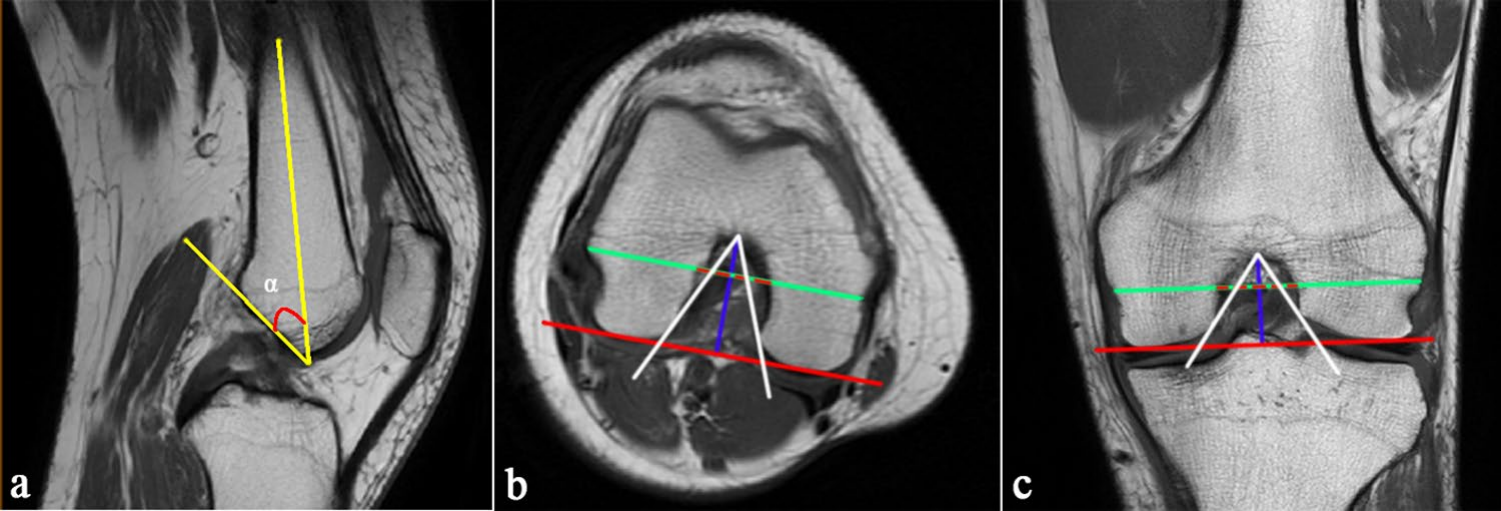
Morphological measurements of the femoral condyle. **a** Sagittal MRI image of the knee, showing the ‘*α*’ angle measurement. **b** Axial MRI image of the knee. The solid red line represents the distal end of the femoral condyles. The blue line represents the intercondylar depth. The green line represents the femoral condylar width. The dashed red line represents the intercondylar notch width. The intercondylar is measured between the two white lines. **c** Coronal MRI image of the knee. The measurement methods are same as **b**

**Fig. 2:**
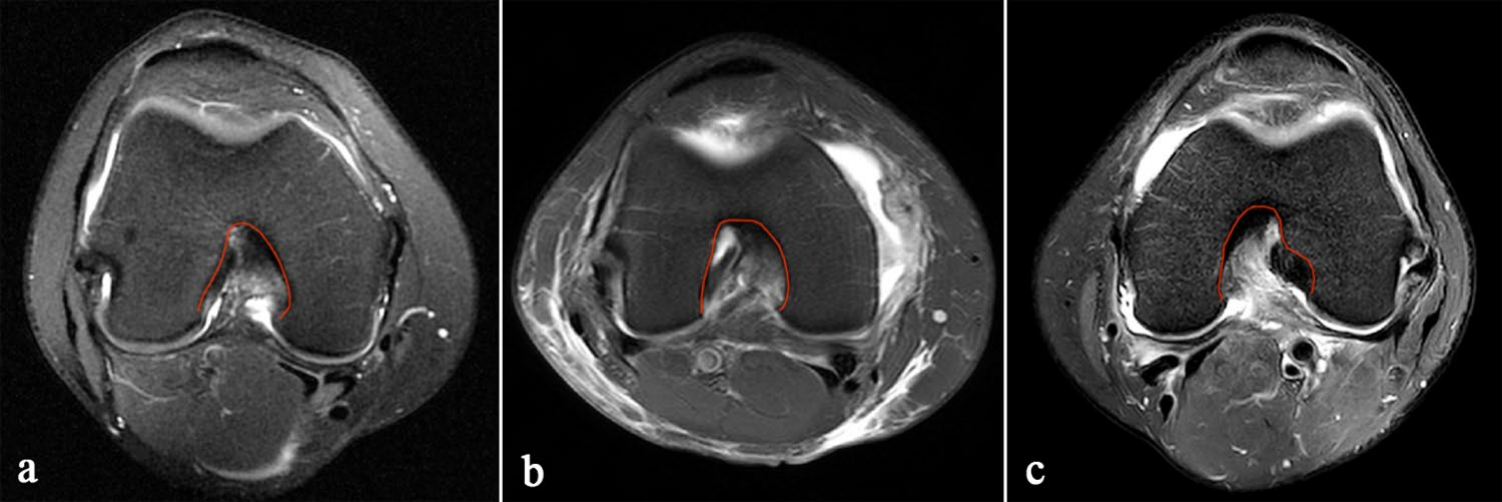
Axial MRI images of the knee, showing the three different intercondylar shape. **a** Type A. **b** Type U. **c** Type W

The images were obtained from our hospital’s Picture Archiving and Communication System (PACS). Measurement accuracy of the system is 0.01 mm. On the sagittal image, the section where the entire Blumensaat line (BL) can be seen was identified. In this section, the ‘α’ angle was measured between the BL and the long axis of the femur (Fig. 1a) [1, 2, 5]. Then, the intercondylar notch width, femoral condylar width, and intercondylar notch angle were measured in both the axial image and coronal image. The intercondylar notch width and the femoral condylar width were measured on the same plane, which was parallel to the plane of the distal end of the femoral condyles and located at the upper 1/3 of the intercondylar depth. The intercondylar depth is the vertical distance between the highest point of the femoral notch and the plane of the distal end of the femoral condyles. Herein, the plane of the distal end of the femur was represented by the line tangent to the lowest point of the cartilage surface of the medial and lateral condyles. The intercondylar notch angle was measured between 2 lines drawn from the highest point of the femoral notch to the medial and lateral margins of the intercondylar notch (Fig. 1b, c) [11, 21]. The NWI is the ratio of the intercondylar notch width to the femoral condylar width. Finally, three types of intercondylar notch shapes (types A, U, and W) were analysed on the axial image. Type A was defined as the intercondylar fossa gradually narrowing from the bottom to the top in the axial MRI image. Type U with the intercondylar fossa has a flatter and wider top than Type A. Type W is a special Type U with two vertices of intercondylar fossa (Fig. 2) [1, 5, 19].

Twenty patients (ten PCL rupture patients and ten control group patients) were randomly selected and analysed by two trained orthopaedists to ensure sufficient interobserver reliability of the measurement methods [6]. The intraclass correlation coefficient (ICC) (2,1) was used to evaluate the measurement results between the observers.

### Statistical analysis

All experimental data (continuous variables) are expressed as the means and standard deviations (SD) or ranges. A paired-sample *t* test was used to compare differences in mean intercondylar notch width, femoral condylar width, NWI, and the intercondylar notch angle from the axial and coronal images and the mean ‘*α*’ angle value between the case and control groups. The Chi-squared test was used to determine the differences in the proportions of the three intercondylar notch types between the two groups. *P* < 0.05 was considered statistically significant. All statistical analyses were performed using SPSS software (IBM SPSS Statistics for Windows, IBM Corp, Armonk, NY, Version 19.0). Given α err prob value (0.05), sample size, and effect size, post hoc analysis was conducted to calculate the statistical power of the study using G*Power 3.1.9.4. A power value > 0.8 was considered indicative of adequate statistical power.

## Results

The mean (range) interobserver ICC was 0.9 (0.7–1.0), indicating that all the measurements agreed well between the observers. The morphological characteristics of the two groups of females and males are presented in Tables 1 and 2, respectively.

**Table 1:** Comparison of the morphological characteristics of the study groups of the females

	PCL rupture group (*n* = 41)	Control group (*n* = 41)	*P *value
*α* angle (°)	37.2 ± 5.6	35.2 ± 5.9	n.s
Axial intercondylar notch width (mm)	17.2 ± 2.0	17.7 ± 1.6	n.s
Axial femoral condylar width (mm)	66.4 ± 3.8	66.1 ± 3.1	n.s
Axial NWI	0.3 ± 0.03	0.3 ± 0.03	n.s
Axial intercondylar notch angle (°)	50.0 ± 7.1	51.4 ± 8.6	n.s
Coronal intercondylar notch width (mm)	17.2 ± 1.7	16.3 ± 2.1	**0.03**
Coronal femoral condylar width (mm)	66.3 ± 2.8	67.2 ± 2.6	n.s
Coronal NWI	0.3 ± 0.02	0.2 ± 0.03	**0.01**
Coronal intercondylar notch angle (°)	56.2 ± 8.2	54.3 ± 6.3	n.s
Intercondylar notch shape			
A	20 (48.8%)	21 (51.2%)	n.s
U	17 (41.5%)	17 (41.5%)	
W	4 (9.8%)	3 (7.3%)	

Bold indicates statistical significance (*p* < 0.05)

**Table 2:** Comparison of the morphological characteristics of the study groups of the males

	PCL rupture group (*n* = 62)	Control group (*n* = 62)	*P* value
*α* angle (°)	37.0 ± 6.4	37.4 ± 6.1	n.s
Axial intercondylar notch width (mm)	19.7 ± 1.8	19.6 ± 1.8	n.s
Axial femoral condylar width (mm)	76.5 ± 3.8	77.6 ± 4.2	n.s
Axial NWI	0.3 ± 0.02	0.3 ± 0.02	n.s
Axial intercondylar notch angle (°)	51.6 ± 7.3	51.5 ± 7.1	n.s
Coronal intercondylar notch width (mm)	19.1 ± 2.1	19.1 ± 2.3	n.s
Coronal femoral condylar width (mm)	76.4 ± 3.6	78.4 ± 3.3	**0.001**
Coronal NWI	0.2 ± 0.03	0.2 ± 0.03	n.s
Coronal intercondylar notch angle (°)	57.4 ± 7.8	56.8 ± 8.3	n.s
Intercondylar notch shape			
A	35 (56.5%)	41 (66.1%)	n.s
U	19 (30.6%)	14 (22.6%)	
W	8 (12.9%)	7 (11.3%)	

Bold indicates statistical significance (*p* < 0.05)

In the female population, the difference in the mean coronal intercondylar notch width (PCL rupture group: 17.2 ± 1.7 vs. control group: 16.3 ± 2.1 mm, *P* = 0.03) was significantly different between the groups. The difference in the mean coronal NWI (PCL rupture group: 0.3 ± 0.02 vs. control group: 0.2 ± 0.03, *P* = 0.01) was significantly different between the groups. The authors found no significant differences in the mean intercondylar notch widths, femoral condylar widths, NWI, and intercondylar notch angles of the axial image; the mean femoral condylar widths, and intercondylar notch angles of the coronal image; the mean ‘α’ angle values; and the differences in the proportion of three intercondylar notch types between the PCL rupture group and control group in the female population (n.s.) (Table 1).

In the male population, the difference in the mean coronal femoral condylar width (PCL rupture group: 76.4 ± 3.6 vs. control group: 78.4 ± 3.3 mm, *P* = 0.001) was statistically significant between the groups. The differences in the mean intercondylar notch width, femoral condylar width, NWI, and intercondylar notch angle on axial images, the mean intercondylar notch width, NWI, and intercondylar notch angle on coronal images, the mean ‘*α*’ angle, and the difference in the proportion of three intercondylar notch types were not significantly different between the study groups (n.s.), (Table 2).

## Discussion

The most important finding of this study was that an increased coronal NWI is a risk factor for PCL rupture in the female population, and decreased condylar width is a risk factor for PCL rupture in the male population. Although the difference in the mean coronal intercondylar notch width was statistically significant between the study groups in the female population, we are not able to conclude that the increased coronal intercondylar notch width is a risk factor for PCL rupture in the female population with a power value < 0.8.

Van Kuijk et al. first examined the morphological characteristics of patients with PCL rupture. They used statistical shape modelling software and reported that a sharper intercondylar notch and flatter tibial eminence are risk factors for PCL rupture [22]. Although the study was ground-breaking, some limitations should be noted. The most critical point is that it did not analyse gender factors separately; it is well known that females and males exhibit different skeletal characteristics. The lack of separate analyses might explain why the results of the present study differed from those of the above study. The authors found that in both the female and male populations, the difference in notch angle between the PCL rupture group and the control group was not statistically significant (Tables 1, 2). Fan et al. reported that coronal NWI is related to PCL avulsion fracture in the female population [8]. PCL avulsion fracture is a special type of PCL injury, and the inconsistency between the present study results and those of Fan et al. might be attributed to a variety of factors. First, age might represent one of the factors. Human skeletal characteristics might change with age. The mean age was 54 years in Fan’s study group. However, the mean age was 39 years in the PCL rupture group in this study. Finally, racial differences might be responsible for the differences between the two studies. Fan’s research participants were individuals from northern China, whereas research participants in the present study were from southern China. Differences in skeletal morphological characteristics are noted among individuals from different regions of China [15, 23].

The incidence of PCL rupture is low. Therefore, there are limited studies on the morphological risk factors for PCL rupture. To the best of the authors’ knowledge, this is the first study to evaluate the relationship between the ‘α’ angle, femoral condylar width, intercondylar notch angle, intercondylar notch shape and PCL rupture. The difference in the mean coronal NWI of the female population was statistically significant between the study groups. This finding indicates that patients with a PCL rupture may not have a sharper intercondylar notch angle. This is a surprising finding because a sharper intercondylar notch angle is generally considered a risk factor for ACL. The difference in coronal femoral condylar width between the study groups was statistically significant in the male population. Although the difference in axial condylar width was not statistically significant, the condylar width of the PCL rupture was also smaller than that of the control group (Table 2). In this study, the mean femoral condylar width in the PCL rupture group in the male population was 1.1 mm and 2.0 mm smaller than that of the control group on the axial and coronal images, respectively.

The present study had some limitations. First, the sample size was small given the low incidence of PCL rupture, especially in females. Some patients were suspicious of PCL rupture on MRI but were not finally diagnosed due to conservative treatment and without arthroscopy. They were not included in the study, which may lead to a reduction in sample size. The age in the study groups was widely distributed, and the knees exhibited degeneration with ageing. Furthermore, the injury mechanism in all the patients in the PCL rupture group was not the same.

## Conclusions

A larger coronal NWI was the greatest risk factor of PCL rupture in the female population. In the male population, decreased coronal condylar width was the greatest risk factor of PCL rupture. The results of the study did not indicate that patients with a PCL rupture have a stenotic intercondylar notch.

## Declarations

### Data availability

All data used in this study were collected from MRI of 206 human subjects. The dataset in this article is available from the corresponding author on reasonable request.

## References

[1] Barnum MS, Boyd ED, Vacek P, Slauterbeck JR, Beynnon BD (2021). Association of geometric characteristics of knee anatomy (alpha angle and intercondylar notch type) with noncontact ACL injury. Am J Sports Med.

[2] Bayer S, Meredith SJ, Wilson KW, de Sa D, Pauyo T, Byrne K, McDonough CM, Musahl V (2020). Knee morphological risk factors for anterior cruciate ligament injury: a systematic review. J Bone Jt Surg Am.

[3] Bernhardson AS, Aman ZS, Dornan GJ, Kemler BR, Storaci HW, Brady AW, Nakama GY, LaPrade RF (2019). Tibial slope and its effect on force in anterior cruciate ligament grafts: anterior cruciate ligament force increases linearly as posterior tibial slope increases. Am J Sports Med.

[4] Bernhardson AS, DePhillipo NN, Daney BT, Kennedy MI, Aman ZS, LaPrade RF (2019). Posterior tibial slope and risk of posterior cruciate ligament injury. Am J Sports Med.

[5] Bouras T, Fennema P, Burke S, Bosman H (2018). Stenotic intercondylar notch type is correlated with anterior cruciate ligament injury in female patients using magnetic resonance imaging. Knee Surg Sports Traumatol Arthrosc.

[6] Dare DM, Fabricant PD, McCarthy MM, Rebolledo BJ, Green DW, Cordasco FA, Jones KJ (2015). Increased lateral tibial slope is a risk factor for pediatric anterior cruciate ligament injury: an MRI-based case-control study of 152 patients. Am J Sports Med.

[7] DePhillipo NN, Zeigler CG, Dekker TJ, Grantham WJ, Aman ZS, Kennedy MI, LaPrade RF (2019). Lateral posterior tibial slope in male and female athletes sustaining contact versus noncontact anterior cruciate ligament tears: a prospective study. Am J Sports Med.

[8] Fan N, Zheng YC, Zang L, Yang CG, Yuan S, Du P (2021). What is the impact of knee morphology on posterior cruciate ligament avulsion fracture in men and women: a case control study. BMC Musculoskelet Disord.

[9] Gwinner C, Weiler A, Roider M, Schaefer FM, Jung TM (2017). Tibial slope strongly influences knee stability after posterior cruciate ligament reconstruction: a prospective 5- to 15-year follow-up. Am J Sports Med.

[10] Hodel S, Kabelitz M, Tondelli T, Vlachopoulos L, Sutter R, Fucentese SF (2019). Introducing the lateral femoral condyle index as a risk factor for anterior cruciate ligament injury. Am J Sports Med.

[11] Jha V, Pandit A (2021). Notch volume measured on magnetic resonance imaging is better than 2-dimensional notch parameters for predicting noncontact anterior cruciate ligament injury in males. Arthroscopy.

[12] Kızılgöz V, Sivrioğlu AK, Aydın H, Ulusoy GR, Çetin T, Tuncer K (2019). The combined effect of body mass index and tibial slope angles on anterior cruciate ligament injury risk in male knees: a case-control study. Clin Med Insights Arthritis Musculoskelet Disord.

[13] Kwak YH, Nam JH, Koh YG, Park BK, Kang KT (2021). Anatomic differences in the sagittal knee joint are associated with ACL injury: results from a skeletally immature Korean population. Orthop J Sports Med.

[14] Li Z, Li C, Li L, Wang P (2020). Correlation between notch width index assessed via magnetic resonance imaging and risk of anterior cruciate ligament injury: an updated meta-analysis. Surg Radiol Anat.

[15] Liu T, Wang CY, Xiao JL, Zhu LY, Li XZ, Qin YG, Gao ZL (2014). Three-dimensional reconstruction method for measuring the knee valgus angle of the femur in northern Chinese adults. J Zhejiang Univ Sci B.

[16] Longo UG, Viganò M, Candela V, de Girolamo L, Cella E, Thiebat G (2021). Epidemiology of posterior cruciate ligament reconstructions in Italy: a 15-year study. J Clin Med.

[17] Loughran GJ, Vulpis CT, Murphy JP, Weiner DA, Svoboda SJ, Hinton RY, Milzman DP (2019). Incidence of knee injuries on artificial turf versus natural grass in national collegiate athletic association American football: 2004–2005 through 2013–2014 seasons. Am J Sports Med.

[18] Lynch TB, Chahla J, Nuelle CW (2021). Anatomy and biomechanics of the posterior cruciate ligament. J Knee Surg.

[19] Shekari I, Shekarchi B, Abbasian M, Minator SM, Momeni MA, Kazemi SM (2020). Predictive factors associated with anterolateral ligament injury in the patients with anterior cruciate ligament tear. Indian J Orthop.

[20] Shelbourne KD, Clark M, Gray T (2013). Minimum 10-year follow-up of patients after an acute, isolated posterior cruciate ligament injury treated nonoperatively. Am J Sports Med.

[21] Shen X, Xiao J, Yang Y, Liu T, Chen S, Gao Z, Zuo J (2019). Multivariable analysis of anatomic risk factors for anterior cruciate ligament injury in active individuals. Arch Orthop Trauma Surg.

[22] van Kuijk K, Reijman M, Bierma-Zeinstra S, Waarsing JH, Meuffels DE (2019). Posterior cruciate ligament injury is influenced by intercondylar shape and size of tibial eminence. Bone Jt J.

[23] Wang Y, Zeng Y, Dai K, Zhu Z, Xie L (2010). Normal lower-extremity alignment parameters in healthy Southern Chinese adults as a guide in total knee arthroplasty. J Arthroplast.

